# The Effect of Peer Support with Telecommunication on Subjective Well-being in Colorectal Patients: A Randomized Controlled Clinical Trial

**DOI:** 10.30476/ijcbnm.2021.88061.1484

**Published:** 2021-04

**Authors:** Mahla Rahimi, Mehrsadat Mahdizadeh, Hamid Chamanzari, Seyed-Mousa Mahdizadeh

**Affiliations:** 1 Nursing and Midwifery Care Research Center, School of Nursing and Midwifery, Mashhad University of Medical Sciences, Mashhad, Iran; 2 Department of Health Education and Health Promotion, Social Determinants of Health Research Center, School of Health, Mashhad University of Medical Sciences, Mashhad, Iran; 3 Department of Medical-Surgical Nursing, Nursing and Midwifery Care Research Center, School of Nursing and Midwifery, Mashhad University of Medical Sciences, Mashhad, Iran

**Keywords:** Colorectal neoplasms, Mental health, Neoplasms, Social support, Telecommunications

## Abstract

**Background:**

Colorectal cancer has a widespread impact on the psychological and physical dimensions of patients and threatens their subjective well-being. Peer support is an effective strategy to increase subjective well-being in cancer patients. This study aims to evaluate the impact of peer support through telecommunications on the subjective well-being of colorectal cancer patients.

**Materials:**

This randomized clinical trial was conducted on 60 patients with colorectal cancer in Mashhad, Iran from November 2018 to April 2019. Two educational hospitals were selected through random sampling from four educational hospitals. Then, participants were randomly selected from the list of patients in each group using a block randomization method. The intervention group received the peer support program by using telephone and virtual social networks for a month. The data were collected by the Warwick-Edinburgh Subjective Well-being Scale before and after the intervention and were then analyzed through independent t-test, paired t-test, and chi-squared test using SPSS version 16. The level of significant was set at P<0.05.

**Results:**

Before the intervention, the mean subjective well-being score of the patients did not show significant difference in the intervention and control groups, respectively (27.8±5.4 vs. 27.6±6.3, P=0.619). However, after the intervention, the mean subjective well-being score of the intervention group showed a significant increase compared to the control group (49.16±3.3 vs. 26.6±6.1, P<0.001).

**Conclusion:**

This randomized controlled trial shows that peer support interventions through telecommunication can improve the subjective well-being of patients with colorectal cancer. Therefore, this method can be used as an effective palliative approach to promote patients’ subjective well-being. Trail Registration Number IRCT20190123042480N1

## INTRODUCTION

Colorectal cancer is the fourth prevalent cancer and the most important cause of death in the world. Its prevalence is continuously increasing in developing countries. ^1 , 2^ In the last three decades, the prevalence and mortality of this disease have considerably increased in Middle East countries, including Iran. ^3^

Researchers believe that the main cause of mortalities resulting from colorectal cancer is problems related to disability, as well as the dysfunctions associated with the diseases and the treatment and care process. ^4 , 5^ The diagnosis and treatment of cancer often cause many psychological problems that threaten patients’ mental health. ^6^ Cancer patients experience different psychological reactions, including a range of problems related to poor of spirit, passivity, and anger. These problems can affect the psychological and physical dimensions of patients. ^7^ A systematic review study shows that mental health issues expose patients to a wide range of psychological problems, including post-traumatic stress, anxiety, and depression. ^8^ However, patients with good mental health find themselves in control of their lives. They have the ability to control stress and negative emotions, and believe that their living conditions are changeable. Therefore, they want to continue living. ^9^ Mental well-being can lead to more success, supportive social relationships, hopefulness, happier life, longer live, and better health status. ^10 , 11^

Evidence shows that the level of social support decreases following the colorectal cancer.12 Social support is a factor closely tied with mental health. ^13^ Peer group is one such supportive force that can affect the prevention and management of chronic disease. ^14^

Peer support increases cancer patients’ knowledge about their disease, disease adaptation skills, satisfaction, psychological adaptation, and hope, and reduces their stress. ^15^ A successful peer can share her experiences, strengths, and weaknesses with patients at the lowest cost. ^14^ Peer support is a form of social support that can be used for empowering patients for adaptation to the disease. Peer volunteers have a better understanding of the client’s situation and cooperate with patients and healthcare providers to resolve obstacles in the healthcare system, and overcome other practical challenges such as childcare, transportation, and communication problems. ^16^

Results of a study showed that peer education could reduce depression in patients with chronic disease. ^17^ A systematic review study showed that the type of intervention based on peer support has different effects on the outcomes of breast cancer. This study also shows that internet-based peer support has no significant impact on the outcomes in patients with breast cancer. ^18^ On the other hand, the researchers state that peer support interventions through telecommunication including the use of telephone, mobile, and internet, can improve the effectiveness of chronic diseases treatment. Also, this support method is available and cost-effective, so it is an appropriate method of delivering information and experience to patients. Based on studies, technology-based peer support interventions can be a satisfying alternative to face-to-face peer interaction. ^19 , 20^

It has been shown that online group support compared to face-to-face group support can better affect the decision-making of patients with prostate cancer. ^21^ Although many studies have discussed the effects of peer education on patient with chronic disease, ^16 , 22^ the effect of telecommunication-based peer support using telephone and virtual social networks is not well specified yet. ^23^ This study aimed to evaluate the effect of peer support with telecommunication on the subjective well-being of patients with colorectal cancer.

## MATERIALS AND METHODS

This double-blinded randomized clinical trial with two groups (intervention and control) was conducted in Mashhad, Iran from November 2018 to April 2019. The research population comprised all the patients with colorectal cancer visiting the educational hospitals of Mashhad University of Medical Sciences, Mashhad, Iran. 

The sample size was calculated by conducting a pilot experiment (as no similar study was available) on 20 patients (10 subjects in each group), apart from the sample of the present study, and by comparing the mean of two independent populations. Based on the mean and SD of subjective well-being in the two groups after a two-week follow-up in the pilot study, and by using α=0.05, and β=0.20, the sample size was estimated at 22 subjects in each group (The following formula). To ensure the sufficiency of the sample size, a sample of 30 subjects in each group (60 in total) was included. 

$n=\frac{{\left(Z_{\mathrm{1-\alpha /2}}+Z_{\mathrm{1-\beta}}\right)}^{2}\times [(S_{1}^{2}+S_{2}^{2})]}{{\left(X_{1}-X_{2}\right)}^{2}}$

$n=\frac{{\left(1.96+0.84\right)}^{2}\times [(1.20^{2}+1.16^{2})]}{{\left(3.3-4.3\right)}^{2}}=22$

**Figure 1:**
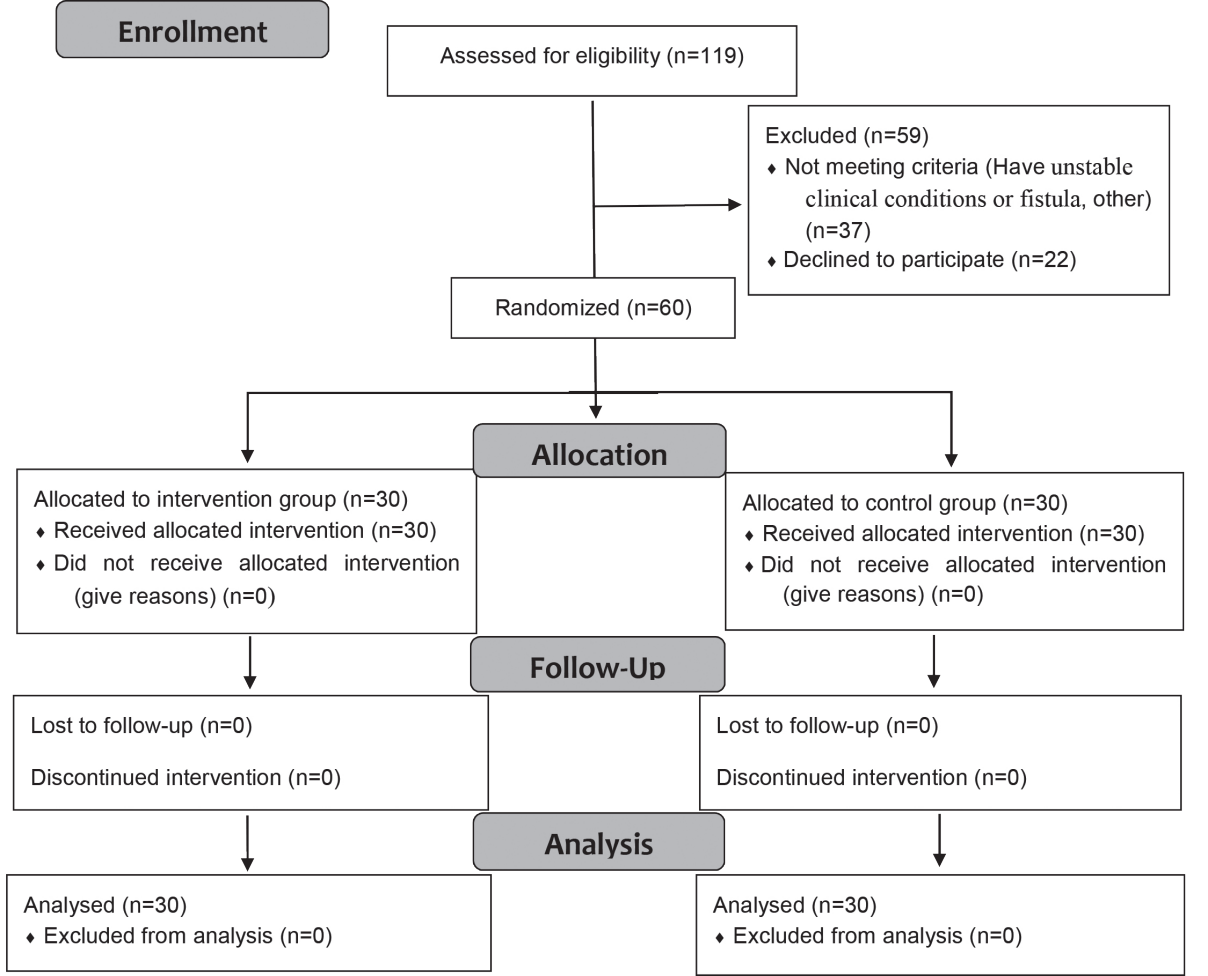
CONSORT Flow Diagram of Study Sample

In order to select samples, first, 4 educational hospitals were selected from 15 hospitals affiliated to Mashhad University of Medical Sciences in Iran through purposive sampling. These hospitals are educational and governmental hospitals with oncology wards. They also perform the hospitalization, treatment and nursing-care processes for patients with different social and economic status. Then, 2 hospitals were randomly assigned to intervention and control groups from 4 educational hospitals using the flip the coin method. This method of considering the hospital as an intervention group and a control group helps prevent errors that may be caused by the transmission of intervention information between the two groups. Finally, for the selection of patients in the intervention and the control group, from the list of patients in each of these two hospitals according to the eligible criteria (58 patients in hospital A and 61 patients in hospital B), 30 male and female patients were enrolled in the study by block randomization method. In terms of gender (female/male), patients are randomly placed in quadruple blocks in equal proportions (1: 1). Patient allocation continued until two groups of both sexes were equal (Figure 1). 

In order to prevent possible bias, the statistician and peer group that participated in the data analysis and implementation of intervention and data collection, respectively, were blinded to the allocation of the groups. To this end, the intervention and the control group were named with numbers 1 and 3, respectively, and packed in similar matte envelopes. The envelope was prepared by a person who had no role in sampling, performing the intervention and analyzing the data. 

The inclusion criteria were willingness to participate, providing written informed consent for participation, age at least 18 years, having colorectal cancer based on cytological diagnostic findings and confirmed by an oncologist (based on patient files), having minimum literacy, auditory and visual health, willingness to share their telephone number for calls, and the ability to use smartphones. The exclusion criteria were unwillingness to continue participation, returning incomplete questionnaires, not completing the intervention course for any reasons or attention for <75% of the determined amount, not establishing successful telephone and Internet calls, having unstable clinical conditions during the research period, e.g. hemodynamic changes, reduced consciousness level, or the occurrence of fistulas. 

Four patients (2 women and 2 men) who met the criteria for peer entered the study for performing the peer support intervention. These criteria included the previously mentioned criteria for selecting patients, in addition to scoring at least 40 on the Warwick-Edinburgh subjective Well-being Scale and successfully passing the stages of treatment.

The research assistant who was blind to the intervention and control groups collected the data. The data collection instrument was a demographic and disease-related information questionnaire and the Warwick-Edinburgh subjective Well-being Scale. The demographic and disease-related questionnaire included 6 questions on age, sex, marital status, level of education, occupation, insurance, and 5 questions about diseases information, such as type of cancer, stage of disease, history of chemotherapy, ostomy and radiotherapy. The Warwick-Edinburgh Subjective Well-being Scale developed by Tennant et al in 2007 includes 14 questions and examines subjective well-being in the dimensions of positive affect (relaxation, feelings optimism, and cheerfulness), satisfaction with interpersonal relationships, and positive performance (clear thinking, personal development, self-acceptance, energy, autonomy and competence). People who complete the scale are required to mark the box that their experience with each item identifies over the past two weeks. The items are scored on a five-point Likert scale (none of the time, rarely, sometimes, often, all the time) from none of the time (1) to all of the time (5). The minimum and maximum scores are 14 and 70, in that order, with higher scores indicating a higher level of subjective well-being. In the study of Tennant et al, in a sample of population, the result of construct validity (GFI>0.9, AGFI>0.08, RMSEA=0.06, P<0.05) showed that this questionnaire is a valid tool. The Cronbach’s α for items was between 0.78-0.92. ^24^ In the other study of Trousselard et al. In 2016, the construct validity (RMSEA=0.07, χ2 (75)=274.21, P<0.001,CFI=0.92, SRMR=0.04, TLI=0.90) and reliability (Cronbach’s α>0.70) of this instrument were deemed acceptable on different groups of the population. ^25^ Rajabi in 2013 examined the psychometrics of the Warwick-Edinburgh Subjective Well-being Scale on a sample of cancer patients and confirmed its construct validity with 12 items and 3 construct including optimism, energetic and positive relationships with others through confirmatory factor analysis (RMSA=0.000, CFI=1, ACFI=0.89, GFI=0.91, RMR=0.06) and reliability with a Cronbach’s alpha of 0.78. In addition, the results of exploratory factor analysis showed that the three factors explain 45.24% of the total variance of the items of the Warwick-Edinburgh Subjective Well-being Scale. In Rajabi’s psychometric version, items 1, 2, 3,8,10, and 14 belong to the optimism structure, items 5, 6 and 11 belonged to the energetic structure, and items 4, 7 and 9 belonged to the positive relationship with others structure. The minimum score for each item is 1 and the maximum score is 5. ^26^ In the present study, the reliability of the instrument was determined using internal and external reliability methods. For this purpose, a questionnaire was given to 30 patients. Value of Cronbach’s alpha for optimism construct, energetic structure, and positive relationship with others structure was 0.783, 0.741, and 0.748 respectively. Cronbach’s alpha had high internal reliability (α=0.875). Also, the reliability of the test-retest in two weeks was confirmed for the whole questionnaire (r=0.81). 

In this study, the intervention with peer support by using communication technology was implemented in five stages for the intervention group. The control group received the routine nursing care program. After the preliminary stages of the study and receiving the approval of the Ethics Committee of the university, a letter of introduction was received from the Faculty of Nursing and Midwifery and offered to the officials of the hospitals. A workshop session was held for familiarizing the volunteers in the peer group with the research. In this session, the peer volunteers received explanations on the objectives of the study. They also acquired knowledge and skills for providing their experimental knowledge to the intervention group. In this workshop, the peers were familiarized with subjective well-being, ways to establish supportive and strong relationships, and subjective well-being improvement strategies. A psychologist was used to educate the peers. In addition, the educational protocol for improving subjective well-being was developed based on the need of patients identified with open-ended questions in the third session and credible sources. ^27 - 29^ This protocol included methods to recognize abilities and talents, dreams, positive beliefs about life, and problem-solving in the face of problems. Another session was held for familiarizing and establishing relationships with the patients in the intervention and control groups separately, and their written informed consent was obtained. Before the intervention, the demographic and disease-related information form was completed by both groups. 

The intervention group received the support program (for emotional, information, and evaluation dimensions) by the peers. The patients under the intervention got to know peers with whom they had the highest degree of similarity in terms of demographic characteristics, disease conditions, and the clinical stage of the disease (a history of surgery, chemotherapy, radiotherapy, metastasis, chronic diseases, ostomy, etc.) and had successfully passed the stages of treatment, and then the intervention started. Each patient received peer support intervention from his/her peer over the phone and virtual social networks for a month. Each peer communicated with groups of 7 or 8 individually, without any relationship with the other groups. For a follow-up of the intervention over the month, receiving feedback on the communication process, and check the stages of the research, the researcher was in touch with the peers and fully supervised the intervention. One month after the start of intervention and follow-up, the post-test was performed in an in-person session set by the patients in the intervention and control groups, and the data were once again collected by the two questionnaires. Table 1 presents a brief summary of the stages of the intervention.

**Table 1:** Stages of intervention and content of educational strategies

Stage of intervention	Purpose	Procedures	Method
First stage (Pre-intervention, 60 min)	Coordinate to implementation of the intervention.	Informing supervisors and department managers about the purpose and process of the intervention.	Face to face communication.
Second stage (Pre-intervention, 120 min)	Informing peer group about the purpose of the study.	For peer group volunteers, the purpose of the study, benefits, and intervention process were described in detail.	Lecture, Workshop
	Training peer group to perform the intervention.	Practical training was performed to transfer the experimental knowledge of the peer group to the patients.	
		A supportive care protocol was developed based on guidelines and scientific resources and delivered to peers to study and educate patients.	
		Ways of communication between peers and the researcher to guide and answer their questions were identified.	
The third stage (Pre-intervention, 120 min)	Informing study participants about the aim and method of the study.	Explaining the purpose and process of conducting the study in detail.	Lecture, Face to face communication.
		Obtaining informed written consent from patients in the control group and intervention group.	
		Needs assessment of patients’ subjective well-being with open-ended questions.	
The fourth stage (Intervention)	Develop an effective care plan for colorectal patients using peer support method.	Conduct of care program with peer support method via telephone, virtual social network sites.	The peer support program, two times a week by phone and three times a week using virtual social networks (based on patients’ preferences) was implemented for the intervention group.
The fifth stage (Post-intervention)	To asses effect of intervention.	To collect data using questionnaires after one month of follow-up in a face-to-face meeting for intervention and control groups.	Face to face communication.
First stage (Pre-intervention, 60 min)	Coordinate to implementation of the intervention.	Informing supervisors and department managers about the purpose and process of the intervention.	Face to face communication.
Second stage (Pre-intervention, 120 min)	Informing peer group about the purpose of the study.	For peer group volunteers, the purpose of the study, benefits, and intervention process were described in detail.	Lecture, Workshop
	Training peer group to perform the intervention.	Practical training was performed to transfer the experimental knowledge of the peer group to the patients.	
		A supportive care protocol was developed based on guidelines and scientific resources and delivered to peers to study and educate patients.	
		Ways of communication between peers and the researcher to guide and answer their questions were identified.	
The third stage (Pre-intervention, 120 min)	Informing study participants about the aim and method of the study.	Explaining the purpose and process of conducting the study in detail.	Lecture, Face to face communication.
		Obtaining informed written consent from patients in the control group and intervention group.	
		Needs assessment of patients’ subjective well-being with open-ended questions.	
The fourth stage (Intervention)	Develop an effective care plan for colorectal patients using peer support method.	Conduct of care program with peer support method via telephone, virtual social network sites.	The peer support program, two times a week by phone and three times a week using virtual social networks (based on patients’ preferences) was implemented for the intervention group.
The fifth stage (Post-intervention)	To asses effect of intervention.	To collect data using questionnaires after one month of follow-up in a face-to-face meeting for intervention and control groups.	Face to face communication.

The data were coded and analyzed in SPSS version 16 by using descriptive statistics (to summarize the data), Kolmogorov-Smirnov test (to examine the distribution of quantitative variables), and independent t-test, paired t-test, and chi-squared test. The significance level was set at <0.05 and the confidence level was 95%.

The Ethics Committee of Mashhad University of Medical Sciences (IR.MUMS.NURSE.REC 1397.076) has approved this study. All patients were informed about the objectives and stages of the study. They were assured that their information would remain confidential. They were also informed that they could withdrawal the study freely.

## RESULTS

In this study, 60 patients participated in the two groups. Analysis of data based on the independent t-test revealed that no significant difference existed between the intervention and control groups in the mean of age (51.1±8.4 vs. 48.5±8.3 years, respectively) (P=0.245). In both groups, the majority of the patients had non-academic education. Also, the majority of the patients were employed and married. In both groups, a higher number of patients had medical insurance, and a large percentage of the patients had colon cancer. The two groups were homogeneous in terms of the other demographic and medical characteristics before the intervention (Table 2). 

**Table 2:** Sociodemographic and medical variables in the intervention and control group

Variable		Study groups		P value
		Control group n=30 N (%)	Intervention group n=30 N (%)	
Sex	Male	15 (50.0)	15 (50.0)	*>0.99
	Female	15 (50.0)	15 (50.0)	
Married	Married	19 (63.4)	26 (86.7)	*0.103
	Single	4 (13.3)	0 (0.0)	
	Widower	4 (13.3)	3 (10.0)	
	Divorce	3 (10.0)	1 (3.3)	
Employed	Employee	2 (6.7)	3(10.0)	**0.369
	Manual worker	11 (36.6)	11(36.6)	
	Free worker	9 (30.0)	13 (43.4)	
	Unemployed/ housewife	6 (20.0)	1 (3.3)	
	Retired	2 (6.7)	2 (6.7)	
Literacy	Less than diploma	20 (66.7)	25(83.3)	*0.582
	Diploma	6 (20.0)	3(10.0)	
	Undergraduate and Bachelor	4 (13.3)	2(6.7)	
Income	Less than adequate	13 (43.3)	18(60.0)	**0.284
	Sufficient	16(53.4)	10 (33.3)	
	More than adequate	1 (3.3)	2 (6.7)	
Housing	Yes	24 (80.0)	28 (93.3)	***0.254
	No	6 (20.0)	2 (6.7)	
Insurance	Yes	25 (83.3)	27(90.0)	**0.706
	No	5 (16.7)	3(10.0)	
Stage of disease	0	4(13.30)	1(3.30)	**0.931
	1	16 (53.30)	22 (73.39)	
	2	8 (26.70)	5 (16.71)	
	3	2 (6.70)	1 (3.30)	
	4	0 (0.00)	1 (3.30)	
Type of cancer	Colon	18 (60.0)	21 (70.0)	**0.417
	Rectum	12 (40.0)	9 (30.0)	
Chemotherapy	Yes	25 (83.3)	30(100.0)	***0.052
	No	5 (16.7)	0 (0.00)	
Radiotherapy	Yes	18 (60.0)	24 (80.0)	***0.113
	No	12 (40.0)	6 (20.0)	
Ostomy	Yes	7 (23.3)	1 (3.3)	***0.053
	No	23 (76.7)	29 (96.7)	

* Chi-square test,

** Fischer exact test,

*** Mann-Whitney U test

According to the normal distribution of variables, parametric tests were used. Before the intervention, the mean of the subjective well-being score of the patients was 27.8±5.4 in the intervention group and 27.6±6.3 in the control group. This difference was not significant based on independent t-test (P=0.619). After the intervention, the mean of the subjective well-being score of the patients was 49.16±3.3 in the intervention group and 26.6±6.1 in the control group. This difference was significant based on the independent t-test (P<0.001). In the inter-group comparison, the paired-t test in the intervention group showed a significant difference in the scores of subjective well-being before and after the intervention (P<0.001). However, the paired t-test did not show a significant difference in the control group (P=0.189). The mean scores of energetic structures, positive relationships with others and optimism of the subjective well-being questionnaire were significantly different in the intervention group (P<0.001) compared to the control group after the intervention. While, before the intervention, this difference of scores in the structures of the questionnaire was not significant (Table 3).

**Table 3:** The inter and intra group comparison of subjective well-being score in the intervention and control groups before and after intervention

Variable	Study groups	Stage of intervention		*P value
		Before mean±SD	After mean±SD	
Subjective well-being	Intervention	27.8±5.4	49.16±3.3	<0.001
	Control	27.6±6.3	26.6±6.1	0.189
**P value		0.619	<0.001	
Energetic	Intervention	6.2±2.0	11.83±1.2	<0.001
	Control	6.0±1.6	6.1±1.3	0.627
**P value		0.439	<0.001	
Positive relationship with others	Intervention	7.4±1.7	12.90±1.02	<0.001
	Control	7.9±1.5	7.2±1.5	0.079
**P value		0.540	<0.001	
Optimism	Intervention	14.3±3.7	24.4±1.7	<0.001
	Control	14.4±3.8	13.3±3.7	0.216
**P value		0.466	<0.001	

* Paired t-test

** Independent t-test,

## DISCUSSION

This study examined the effect of peer support intervention on the subjective well-being of patients with colorectal cancer. After the intervention, the patients’ subjective well-being score and its constructs were significantly higher in the intervention group than the control group. These results demonstrate that and trained peer support with telecommunication affects subjective well-being among patient with colorectal cancer. Although there is little evidence of adverse effects of peer support in cancer patients, findings of a systematic review study showed that interventions performed with online peer support and without trained peers do not have a positive effect on patients and may even have adverse effects on side effects such as depression, anxiety, and quality of life of patients. ^18^ In line with the present study, data from a peer-based intervention using the telephone to reduce distress in women with breast cancer showed that the intervention was significantly effective in reducing their information needs and distress. ^30^

The results of the present study showed that peer support based on telecommunications can improve optimism, positive relationships with others and being energetic. Consistent with the present findings, a study shows that the cancer patients’ hopefulness increases with the increases in their perceived social support. ^31^ The previous findings also reveal that declining quality of social support is an important predictor of increased stress, depression, and negative emotions in breast cancer patients. ^32^ Contrary to present findings, in a cross- sectional study reported that there was no association between hope and support family and community social and the level of suffering of patients with colorectal cancer. ^33^ Various factors can cause this difference in findings, including differences in sample size and the method of conducting the two studies. In line with the study, another study reported that optimism is significantly related to fewer depressive symptoms and anxious, less hopelessness, and better quality of life in patients with cancer. ^34^ Optimistic beliefs about the disease can not only reduce cancer mortality, but also increase the health-related quality of life in cancer patients. ^35^ On the other hand, a study indicates that optimism and hope are not associated with survival in patients with advanced colorectal cancer. ^36^ These conflicting results are likely due to differences in level of sever of illness, questionnaires and the methodological design of the studies.

A systematic review showed that Internet-based educational interventions can mitigate the psychological consequences of cancer, including depression and fatigue, and enhance the patients’ quality of life and mental health. ^18^ However, In another study showed that despite the significant increase in the self-efficacy of cancer management between the experimental and control groups, no significant increase was observed between the two groups in terms of anxiety, depression, and psychological adaptation. ^37^ This result is not consistent with the result of the present study. The reason for this difference could be the difference in the study sample and part of the method; in their study, peer support was offered in-person, but in the present study, it was based on communication technology in which there is a higher chance of access to continuous support, with more and better effects on patients’ subjective well-being. ^38^

One of the limitations of the present study was not examining the persistence of the effect of distant peer education for more than one month; if this was done over longer intervals after the intervention, a better judgment could be made as to the effects of this educational method. Another limitation was not paying attention to personal differences in implementing the educational intervention, which could have improved its effectiveness. 

What sets this study apart from other studies is the existence of a peer support program that reduces patient care challenges by using available telecommunication methods. Also, the use of diverse educational content required by patients in each part of the training is another advantage of this research. 

## CONCLUSION

The results of this study indicate the positive effect of peer support through telecommunication on the subjective well-being of patients with colorectal cancer. This supportive method can be implemented with limited resources for improving the subjective well-being and peace of mind of cancer patients. The use of this method in oncology wards can encourage the cooperation of patients with the disease management process. Thus, it is recommended that this methods based on the advancement of technology be used for improving the mental well-being of patients with colorectal cancer, and healthcare workers, especially nurses in oncology wards, be empowered for providing palliative and novel care approaches. Further Interventional studies are useful for assessing patient satisfaction, and the cost-effectiveness of supportive care programs using new communication technologies in other cancer patients.
